# Sub‐Nanometer Ferroelectric Tunnel Junctions With Record‐High on‐Current Density Through Synergistic Microwave Annealing and High‐Field Activation

**DOI:** 10.1002/smll.73730

**Published:** 2026-05-10

**Authors:** Laeyong Jung, Hojung Jang, Jongseon Seo, Hyunsang Hwang

**Affiliations:** ^1^ Center For Single Atom‐Based Semiconductor Device and the Department of Materials Science and Engineering Pohang University of Science and Technology Pohang Republic of Korea

**Keywords:** cross point array applications, ferroelectric device based on hafnium oxide, ferroelectric tunnel junctions, high‐density memory, ultrathin film

## Abstract

High‐density memory systems require a high on‐state current density (J_on_) to ensure fast read speeds and large sensing margins in cross‐point array applications. However, achieving high J_on_ in atomically thin ferroelectric tunnel junctions (FTJs) remains an unmet challenge, hindered by parasitic interfacial layer (IL), depolarization fields, and phase instability that favors the non‐ferroelectric tetragonal phase. Here, a record‐high J_on_ operation in sub‐nanometer FTJs is enabled by combining aggressive device area scaling with low‐temperature microwave annealing. This approach effectively suppresses IL growth, reducing the IL thickness from 0.94 to 0.41 nm, and simultaneously enables a robust orthorhombic phase transition under high electric fields (>15 MV cm^−1^) without dielectric breakdown. By leveraging interface engineering and extreme area scaling, ∼0.9 nm FTJ achieves a record‐high J_on_ exceeding 10^5^ A cm^−^^2^ at low voltage (∼0.4 V) while maintaining a stable on/off ratio (>15). This strategy also induces exceptional endurance (>10^8^ cycles) and retention (projected ∼10 years at 85°C), attributed to the reduced depolarization fields. Furthermore, simulations based on experimental data provide guidelines for achieving ideal FTJ performance at the ultimate scaling limits. Our comprehensive study establishes a clear pathway toward reliable FTJ memory suitable for high‐density array applications without additional material complexity.

## 1 Introduction

The rapid development of big data technologies and artificial intelligence has exposed fundamental limitations of conventional memory architectures, resulting in data‐transfer bottlenecks and excessive power consumption [1, 2, 3]. To mitigate this challenge, there is a pressing need for next‐generation non‐volatile memories that combine fast switching, high density, and energy efficiency. A range of emerging memory technologies has been investigated, including resistive random‐access memory (RRAM) [4, 5, 6], Phase‐Change Memory (PCM) [7, 8]. However, each of these exhibits inherent drawbacks for high‐performance cross‐point array applications. RRAM, which relies on the formation and rupture of conductive filaments, suffers from significant device‐to‐device and cycle‐to‐cycle variability complicating the implementation of reliable multi‐level storage [9]. PCM, while demonstrating robust endurance, typically requires high write and reset current densities for Joule heating, resulting in large energy consumption and serious thermal‐management issues in dense arrays [10].

Among the alternatives, ferroelectric tunnel junction (FTJ) memory has emerged as a particularly promising candidate due to its electric‐field‐driven switching mechanism that enables intrinsically low power operation and fast switching speed [11, 12, 13]. An FTJ is a two‐terminal memristive device in which electrons tunnel through an ultrathin ferroelectric barrier. The polarization state of the ferroelectric layer modulates the tunneling barrier height, yielding distinct high‐ and low‐conductance states which is a phenomenon known as the tunneling electroresistance (TER) effect [14]. In contrast to filamentary‐type memories, FTJ switching is governed by uniform modulation of the energy barrier via an external electric field, rather than localized current‐induced changes. This mechanism results in lower power dissipation, faster switching kinetics, and more uniform switching, highlighting the potential of FTJs for high‐density storage. Early demonstrations of high‐performance FTJs largely employed perovskite ferroelectrics, such as BaTiO_3_ (BTO) and Pb(Zr,Ti)O_3_ (PZT) [15, 16], achieving fast switching speed and multi‐bit storage. Nevertheless, these perovskite‐based FTJs faced integration obstacles due to their incompatibility with silicon‐based CMOS technology. This integration barrier shifted attention toward hafnia‐based ferroelectrics that can be deposited at low temperatures with standard thin‐film techniques.

The discovery of robust ferroelectricity in CMOS‐compatible, doped hafnium oxide (HfO_2_) thin films, in particular Hf_x_Zr_1__−__x_O_2_ (HZO), represented a paradigm shift for the ferroelectric field. HZO has been established as the leading candidate material due to its scalability, relatively low crystallization temperature, large remnant polarization, and manufacturability using mature Atomic Layer Deposition (ALD) techniques [17, 18]. Despite this technological breakthrough, unlocking the full potential of HZO‐based FTJs has been limited by an inevitable scaling dilemma. Achieving a high ON‐state current density (J_o__n_)—often cited as >>100 A cm^−^^2^ for fast and reliable read operations—requires aggressively scaling down the ferroelectric layer thickness. Indeed, if J_on_ is too low, the readout of an FTJ is slow and power‐hungry, and sensing circuits may need extra amplification, complicating high‐density array integration [19]. However, thinning the ferroelectric layer introduces a series of significant physical challenges that paradoxically degrade FTJ performance (Figures S1 and S2). Firstly, at nanometer thickness, an unavoidable and non‐switchable interfacial layer (IL), which is often an oxidized layer at the electrode interface, consumes a significant fraction of the applied voltage and necessitates higher operating electric fields. In ultrathin HZO, this IL becomes disproportionately impactful. It not only reduces the effective field across the ferroelectric but also contributes to a depolarization field (E_dep_) due to incomplete screening of polarization charges. E_dep_ opposes the HZO polarization, destabilizing the remnant polarization states [20]. Notably, IL formation and depolarization are coupled problems: a thicker IL exacerbates E_dep_, and a strong E_dep_ in turn suppresses the switchable orthorhombic (o) phase fraction, dramatically reducing the achievable TER at low bias.

Secondly, in the ultrathin regime (<5 nm), HZO thermodynamically favors the non‐ferroelectric tetragonal (t) phase over the desired ferroelectric o‐phase [21]. This tendency leads to a drastic reduction of the memory window (TER) as thickness decreases. Prior studies attempted to counter this by adding interfacial “seed” layers or dopants to stabilize the o‐phase [22, 23], but such additions come at the cost of increased total thickness or complexity, undermining scalability. Crucially, the most straightforward way to force o‐phase formation, which is applying a strong electric field, has historically been impractical in large‐area ultrathin capacitors due to the high probability of premature dielectric breakdown [24, 25]. This seemingly unavoidable trade‐off between aggressive scaling and ferroelectric performance has thus limited ultrathin FTJ development until now.

In this work, we present a synergistic engineering strategy that directly confronts and overcomes these challenges. The key innovation is the implementation of extreme device area scaling, which dramatically improves the film's tolerance to high electric fields by reducing the statistical likelihood of having a critical defect in the active area. By minimizing defect‐driven weak points, we unlock the ability to apply a robust high‐field wake‐up to the ultrathin ferroelectric, providing the thermodynamic driving force to induce the structural t‐to‐o phase transition, as illustrated in Figure S3. We perform this field‐driven phase engineering in synergy with a low‐temperature microwave annealing (MWA) process, as shown in Figure S4. MWA provides targeted vibrational energy to crystallize HZO at a lower thermal budget than conventional rapid thermal annealing (RTA), thereby suppressing IL growth and actively promoting o‐phase formation during crystallization [26]. Through this combined approach, we demonstrate an HZO FTJ with an effective ferroelectric thickness of ∼0.9 nm that exhibits a record‐high J_on_ (>10^5^ A cm^−^^2^) and a robust TER (>10) at low operating voltage, while also achieving excellent endurance and retention. This comprehensive study not only surpasses previous performance limits for FTJs but also provides a scalable blueprint for integrating ferroelectric memory at the ultimate limits of miniaturization.

## 2 Results and Discussion

**FIGURE 1:**
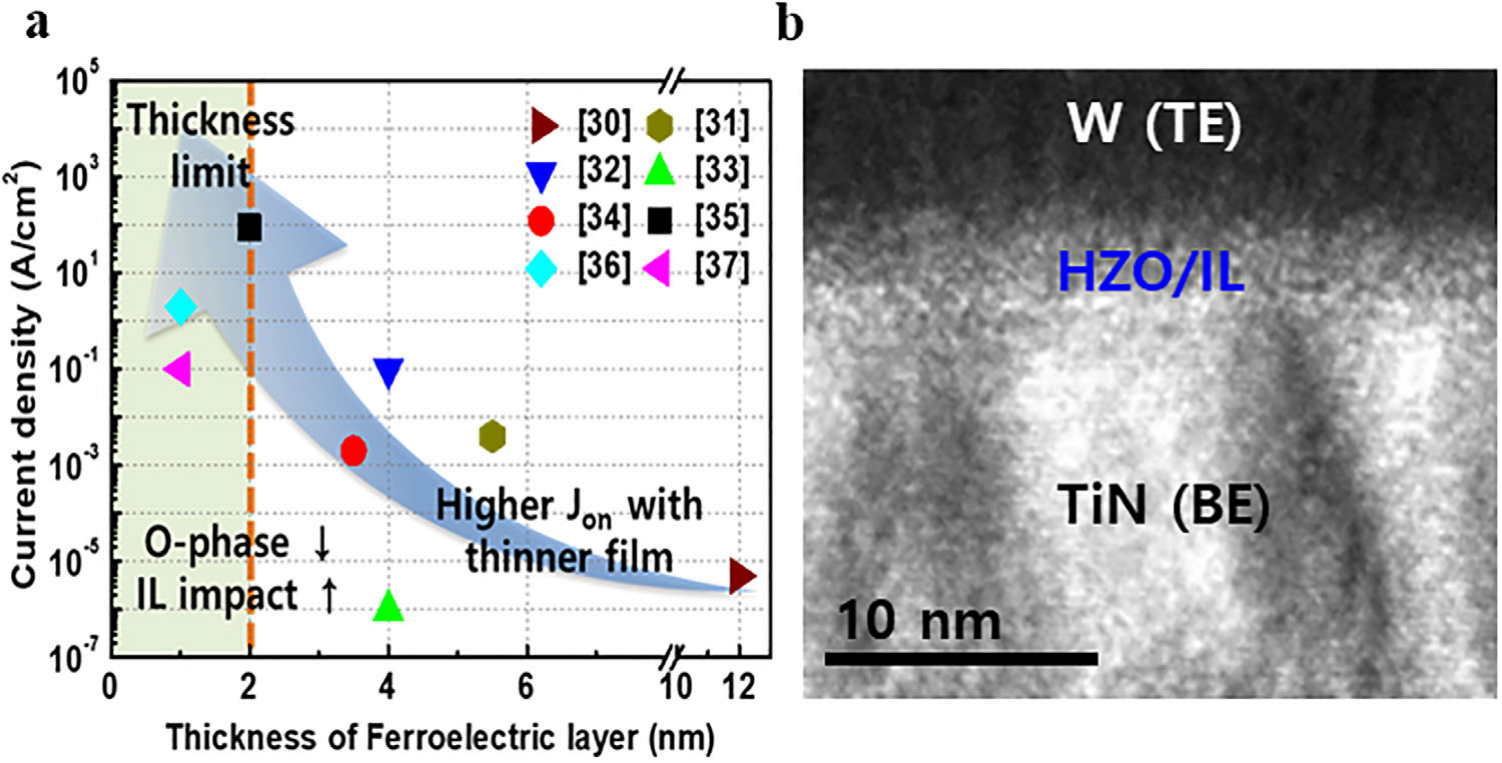
(a) Reported J_on_ of FTJs as a function of ferroelectric‐layer thickness in the literature. Aggressive thinning of HZO has been widely attempted to increase J_on_, but prior studies consistently show a sharp drop‐off in performance below ∼2–3 nm due to reduced o‐phase fraction and increased IL influence. (b) Cross‐sectional HR‐TEM image of a W/HZO/IL/TiN stack, illustrating the ∼1 nm interfacial layer that forms at the HZO/TiN interface during deposition and annealing. This low‐contrast IL is an oxidized layer that consumes part of the applied voltage and contributes to the E_dep_, thereby degrading the FTJ performance in the ultrathin regime.

The intrinsic challenges of scaling HZO‐based FTJs can be understood as a trade‐off between ferroelectric layer thickness and device performance. When HZO films are thinned below ∼10 nm, thermodynamic and interfacial effects make ultrathin HZO films favor the non‐switchable tetragonal (t) phase over the desired orthorhombic o‐phase [27, 28, 29]. This phase instability intensifies as thickness decreases, posing a fundamental barrier to scaling. As shown in Figure 1a, prior reports prevented the attainment of high J_on_ (>100 A cm^−^^2^) due to the dominance of the t‐phase in the ultrathin limit, suppressing ferroelectric polarization and degrading the TER [30, 31, 32, 33, 34, 35, 36, 37]. Since polarization‐driven modulation of the tunneling barrier height is a critical aspect of FTJ operation, the suppression of the o‐phase fraction directly limits device performance in terms of memory window. Conversely, with a higher o‐phase fraction, the potential barrier modulation with the direction of polarization can be enhanced, resulting in higher TER.

In addition to intrinsic phase instability, Figure 1b highlights the presence and significance of the IL that forms between HZO and the TiN electrode. In ultrathin HZO films, the impact of even a sub‐nanometer IL is magnified in terms of voltage drop and E_dep_. Therefore, a larger IL effect reduces J_on_ and compresses TER, whereas a thinner IL reduces E_dep_, improving device performance [20]. To address the impact of IL in the switching layer and stabilize the ferroelectric phase at near‐atomic thickness, the low‐temperature MWA and high electric field induced phase transition were combined as depicted in Figure S5.

The MWA process provides the energy needed for HZO crystallization in a rapid, yet low thermal budget manner. This has two key effects that it facilitates o‐phase formation at a reduced temperature, lowering the kinetic barrier for the o‐phase, and simultaneously limits oxygen vacancy diffusion, thereby suppressing the undesirable chemical reactions that form ILs. Complementarily, the application of a high electric field further drives the t‐ to o‐phase transition in near‐atomic‐thickness HZO. In such near‐atomic‐thickness films, the energy difference between t‐phase and o‐phase is small, and a sufficiently high external field can tip the balance in favor of the polar o‐phase [38, 39, 40, 41]. In essence, the field provides a thermodynamic driving force for phase transformation, analogous to the well‐known “wake‐up” effect but taken to an extreme. By carefully integrating MWA to ensure a clean interface and initial o‐phase nucleation with high‐field application to transform any residual t‐phase regions into the o‐phase, a significant and stable increase in the o‐phase fraction can be achieved. This translates directly into the targeted improvements in both J_on_ and resistance contrast (TER).

**FIGURE 2:**
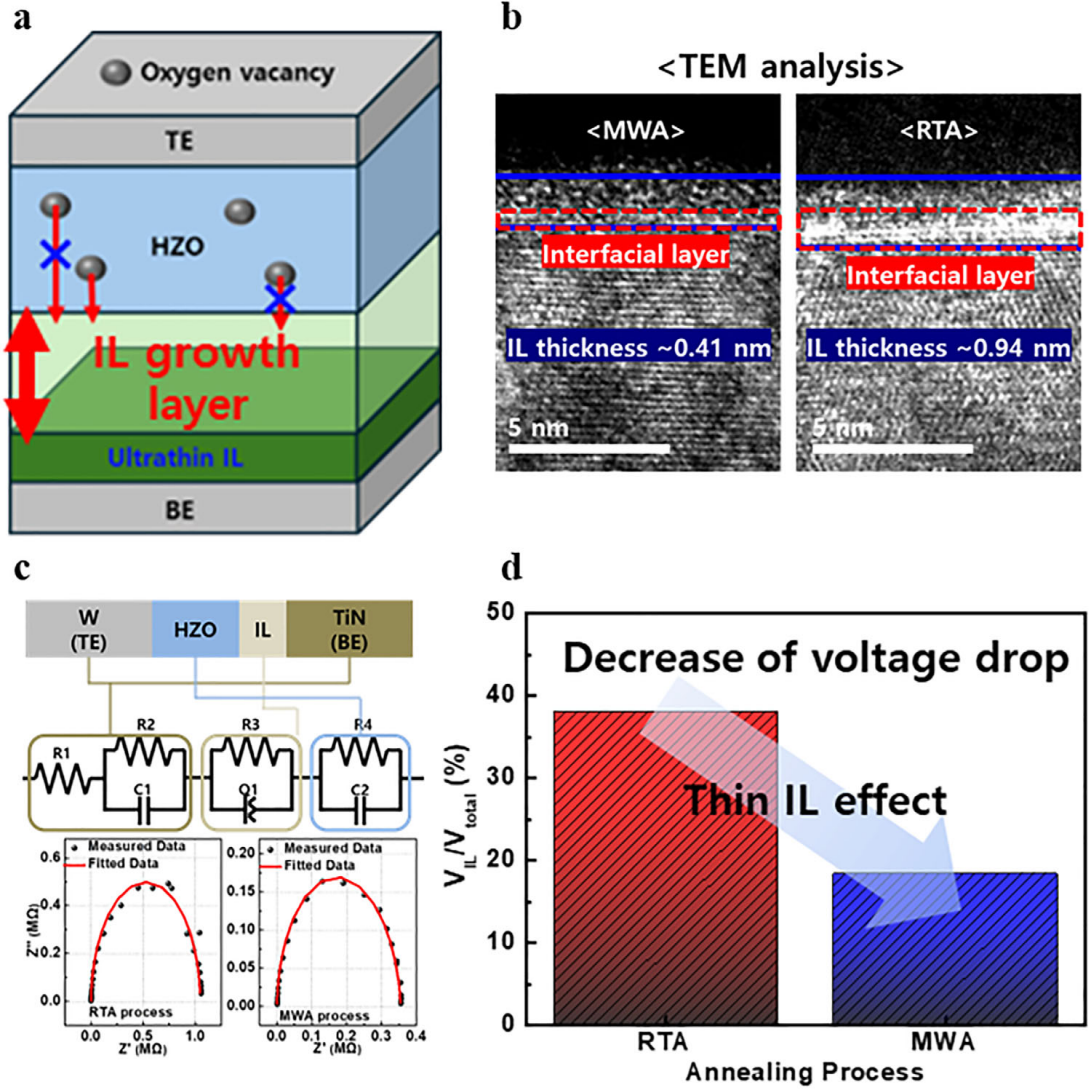
(a) Schematic illustrating the effect of MWA under a low‐thermal‐budget process. Low‐temperature annealing mitigates oxygen vacancy diffusion, thereby suppressing IL growth compared to a conventional high‐temperature RTA. (b) Cross‐sectional HR‐TEM image of MWA‐ and RTA‐treated samples. With suppression of IL growth, The MWA‐treated sample shows a much thinner IL (∼0.41 nm) than the RTA‐treated sample (∼0.94 nm). (c) Simplified equivalent circuit for EIS analysis composed of W/HZO/IL/TiN structure. By fitting the impedance, the resistive and capacitive contributions of the HZO and IL layers can be deconvolved. (d) Voltage division between HZO and IL extracted from EIS measurements. In the RTA‐processed device, ∼38% of the applied voltage drops across the IL, whereas in the MWA‐processed device only ∼18% is lost in the IL. These results indicate that MWA induces thinner IL, leading to the higher J_on_ at the same read voltage.

To mitigate the parasitic IL effect, which is a known challenge with conventional high‐temperature RTA, the low‐thermal‐budget MWA process was employed. The MWA process was carried out at a microwave power of 2 kW, and the annealing time was systematically increased in steps of 10 s to identify the condition that yields the optimal on/off ratio. When the annealing time was too short, the ferroelectric phase was not sufficiently formed, resulting in a limited on/off ratio. In contrast, excessively long annealing led to a significant increase in leakage current, which also degraded the on/off ratio. Based on this optimization, an annealing time of 20 s was selected as the optimal condition and was used throughout this work. In a conventional annealing process, the high thermal budget accelerates the migration of mobile defects, such as oxygen vacancies, toward the interface between HZO and the bottom electrode. This enhanced diffusion promotes undesirable interfacial reactions, most notably oxidation of the metallic electrode, leading to the growth of an IL such as TiO_2_ or TiON. This IL effectively acts as a series capacitor that steals a portion of the applied bias, thereby weakening the electric field across the ferroelectric and diminishing the polarization's influence on the tunneling current [42]. In contrast, MWA introduces a substantially lower thermal budget. As schematically shown in Figure 2a, this distinct heating mechanism drastically reduces the kinetic driving force for atomic diffusion and limits interfacial chemical reactions [43]. Consequently, MWA directly and effectively suppresses the growth of the parasitic IL.

This hypothesis is confirmed by the direct and atomic‐scale evidence from cross‐sectional high‐resolution transmission electron microscopy (HR‐TEM) analysis, as presented in Figure 2b. A comparison between the two samples revealed a remarkable reduction of over 50% in IL thickness, from ∼0.94 nm in the RTA‐treated device to an ultrathin ∼0.41 nm in its MWA‐treated counterpart. Such a pronounced reduction is consistent with diffusion‐limited kinetics expected under low‐thermal‐budget processing and aligns with previous observations that minimizing IL thickness is key to maintaining high TER in FTJs [42, 44].

The electrical impact of a thinner IL is quantified by electrochemical impedance spectroscopy (EIS) analysis (Figure 2c) [45]. First, the impedance was measured with various frequency ranging from 100 Hz to 1 GHz. Subsequently, by fitting the impedance spectra of the W/HZO/IL/TiN stack, the effective resistances of the HZO and IL layers were extracted and the resistance of IL with MWA exhibited low resistance due to the thinner thickness. From these, the voltage drop across each layer under an applied bias was calculated. In the RTA‐treated device, ∼38% of the voltage dropped across the IL, meaning only ∼62% was effective across the HZO. In the MWA‐treated device, the IL accounted for only ∼18% voltage drop, with the vast majority of the bias (∼82%) appearing across the ferroelectric (Figure 2d). This efficient voltage redistribution directly translates to enhanced polarization switching at a given external voltage, boosting J_on_ at the read condition. Furthermore, by minimizing the IL thickness, MWA indirectly mitigates the E_dep_, which opposes remanent polarization and is a primary factor in the retention degradation of ultrathin ferroelectric memories. As a result, the MWA‐induced reduction of E_dep_ leads to more stable polarization states and improved retention, as demonstrated later in the retention tests.

An additional advantage of the MWA approach is its practicality for integration. Unlike some structural interface‐engineering solutions (e.g., inserting buffer layers or complex stacks), which may introduce fabrication complexity or variability across a wafer, MWA is a process‐level improvement [46]. This advantage not only simplifies integration into back‐end‐of‐line process flows but also enables scalability to large wafer sizes without requiring additional optimization of stack composition. Furthermore, the switching layer thickness can be preserved without resorting to additional layer insertions, maintaining both simplicity and compatibility. In summary, MWA provides a high‐quality ferroelectric/electrode interface in our devices. However, interface improvement alone is insufficient to reach the record performance, and stabilizing the ferroelectric phase in sub‐nanometer HZO is also required. This was accomplished by a high‐field activation strategy, which became feasible only after implementing extreme device area scaling, as discussed next.

To leverage the high‐field wake‐up effect for inducing the t‐to‐o phase transition, applying a sufficiently large electric field without dielectric breakdown is a critical prerequisite. In larger devices, the presence of even a single nanoscale defect can initiate breakdown at relatively lower fields, making it extremely challenging to apply fields above ∼10–12 MV cm^−1^ in nanometer‐thick HZO capacitors on the micrometer scale. The breakdown characteristics of our devices were investigated to understand how scaling influences breakdown tolerance.

**FIGURE 3:**
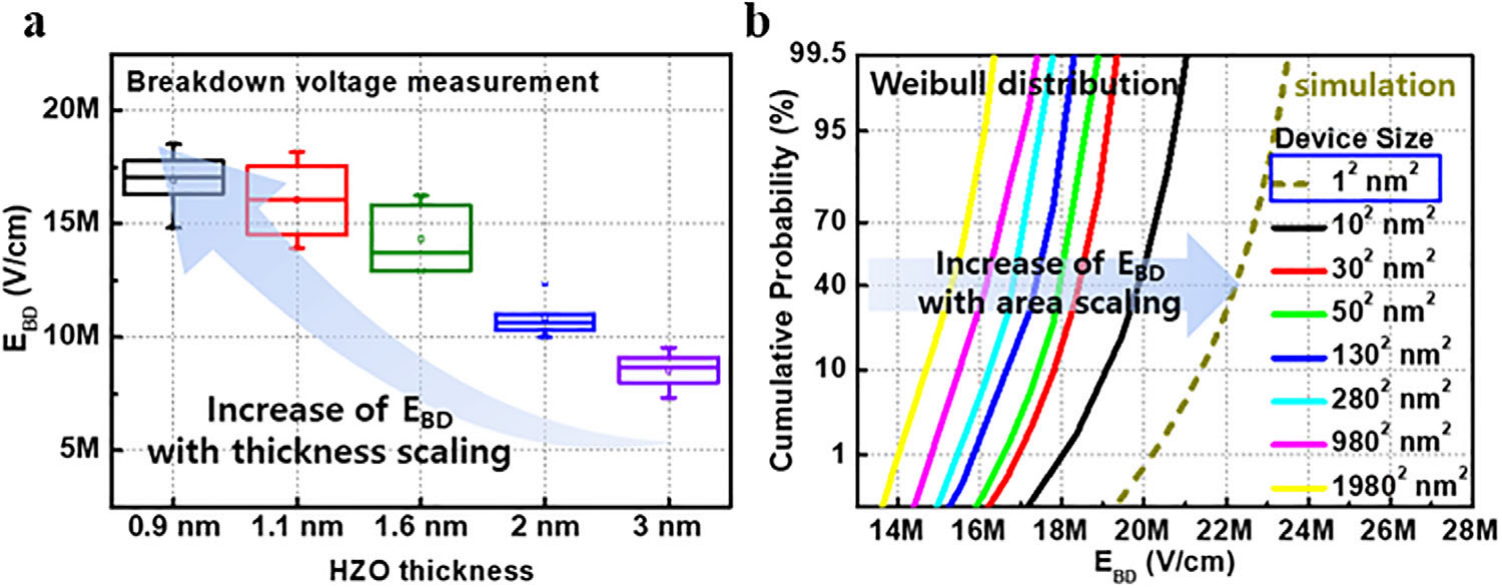
(a) E_BD_ of HZO capacitors as a function of ferroelectric thickness. Thinner HZO films exhibit higher breakdown field values, consistent with the general trend that a thinner dielectric can sustain a larger field before failure. (b) E_BD_ as a function of device area. The device area was measured down to 100 nm^2^ experimentally and extrapolated via Weibull statistical modeling to 1 nm^2^. Smaller area devices show significantly higher E_BD_ due to the reduced probability of a critical defect being present. The simulation (dashed line) suggests that at an extreme area of ∼1 nm^2^, E_BD_ could approach ∼20 MV cm^−1^.

Figure 3a plots the measured breakdown field (E_BD_) as a function of HZO thickness for devices of the same area. As the HZO layer was scaled from 3 nm down to ∼0.9 nm, E_BD_ was observed to increase from ∼7 to ∼16 MV cm^−1^. This trend is consistent with well‐established breakdown models, where thinner dielectric films exhibit higher fields at failure [47].

Even more striking is the effect of device area on breakdown, shown in Figure 3b. E_BD_ for devices was measured across the area ranging from our largest test structures down to the 100 nm^2^ junctions. Empirically, the 100 nm^2^ devices exhibited substantially higher breakdown fields than larger area counterparts. This area‐dependent breakdown behavior is caused by the statistical nature of breakdown: in a larger area, there is a higher chance that a lethal defect or weak region exists somewhere in the dielectric, limiting the overall device E_BD_ [48]. By reducing the capacitor area, most of these defect‐related failure sites were screened out. This can be described by Weibull distribution statistics, which predict a scaling of E_BD_ with volume/area reduction. Our experimental data (solid line) align with this concept, showing E_BD_ shifting to higher fields as the area shrinks. A further probabilistic model was used to extrapolate this trend toward the extreme limit of device area. The model (dashed line) was calibrated with our measurements and a Weibull shape parameter (β) was extracted, yielding β ≈ 41, which this value was extracted from the fitted plot and slope (Figure S6) [49]. This extrapolated data suggests that E_BD_ could approach ∼20 MV cm^−1^ at an area of ∼1 nm^2^.

Collectively, these findings demonstrate that the longstanding breakdown bottleneck of ultrathin FTJs can be effectively overcome by coupling thickness reduction with area scaling. With ∼0.9 nm HZO in a ∼100 nm^2^, a safe high‐field window exceeding 15 MV cm^−1^ was established, which is sufficient to fully induce the ferroelectric o‐phase without causing device failure. Establishing this high‐field window provides a robust physical foundation for reliably switching and retaining polarization in the near‐atomic thickness regime.

**FIGURE 4:**
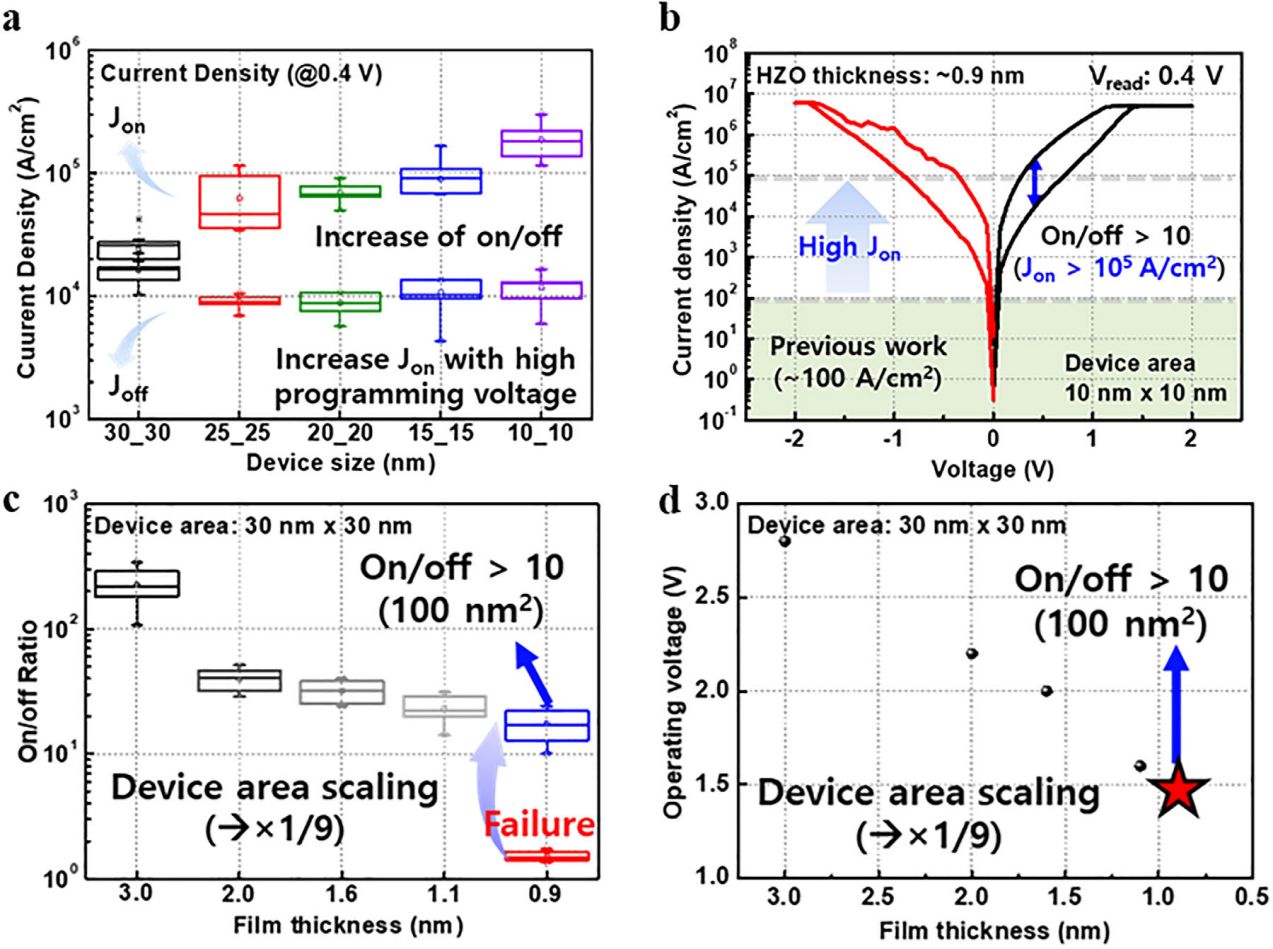
(a) On current density and off current density versus device area at a fixed HZO thickness of ∼0.9 nm. The on/off ratio increases as the device area is reduced, primarily because off‐state current density remains nearly constant with area scaling, whereas on‐state current density increases in smaller devices with higher programming voltage. (b) DC *I–V* characteristics of sub‐1 nm FTJ memory at the device area of 100 nm^2^, showing high J_on_ (>10^5^ A cm^−^^2^) and sufficient on/off ratio (>10) was at low read bias. (c) On/off ratio as a function of HZO thickness for two different device areas (large area ∼900 nm^2^ versus scaled area ∼100 nm^2^). At larger area, the memory window collapses rapidly as HZO thickness approaches 1 nm due to high leakage and early breakdown. In contrast, the scaled 100 nm^2^ devices maintain on/off over 10 even at ∼0.9 nm thickness. (d) Required operating voltage to achieve on/off over 10, as a function of HZO thickness for different device areas. With thinner film thickness, the operating voltage in write process decreased, and FTJ memory at sub‐1 nm showed the lowest operation of 1.5 V.

Building on the previously confirmed increase in field tolerance due to aggressive scaling of both ferroelectric thickness and device lateral area, FTJ operation was investigated under these extreme dimensions. The HZO layer was reduced to ∼0.9 nm, and the junction area was defined at ∼100 nm^2^ by a sidewall process as shown in Figure S7. This extreme scaling enabled us to explore the performance of FTJs in the near‐atomic thickness regime for the first time. As shown in Figure 4a, shrinking the device area improved the memory window at a fixed read bias. The main reason is that the leakage current which determines the off‐state scales roughly with area, indicating that a smaller junction statistically contains fewer defects or conductive paths, thereby suppressing the off‐state current. In contrast, the on‐state dominated by tunneling through the ferroelectric remains relatively unchanged or even improves due to the higher effective field. Furthermore, smaller capacitors can withstand higher electric fields without breaking down, so larger electric fields can be applied to the ferroelectric layer, increasing the wake‐up effect. As a result, the on/off ratio increased with area scaling. This trend accords with the notion that, for a series barrier composed of IL and HZO, the off‐state current is particularly sensitive to defect‐assisted trajectories. Reduced device area statistically suppresses those trajectories and steepens the barrier for the off state.

Figure 4b highlights record performance attained by combining extreme thickness and area scaling. In the sub‐1 nm thickness, the on‐state is governed by direct tunneling through a polarization‐induced barrier. By minimizing the voltage drop across the IL with MWA and maximizing the electric field effect with scaling, high J_on_ (>10^5^ A cm^−^^2^) and stable on/off ratio (>10) were obtained, far exceeding earlier HZO‐based FTJ reports that typically exhibited under 10^2^ A cm^−^^2^ at comparable low biases. Moreover, these results confirm, based on Piezoresponse Force Microscopy measurement, that the observed on/off switching originates from the ferroelectric polarization switching (Figure S8).

Device area scaling also resolves the switching limit of the ultrathin layer. Reliable switching below 1 nm was precluded for devices with an area of 900 nm^2^, and the operating voltage required to attain an on/off ratio over 10 inevitably approached the breakdown threshold. By scaling the area to 100 nm^2^, stable ferroelectric tunneling was observed in the sub‐1 nm regime. Figure 4c shows the trend of on/off ratio versus HZO thickness. Whereas larger area devices showed a rapid loss of read window as the film approaches 1 nm, the 100 nm^2^ devices maintained an on/off ratio over 10 at a thickness of 0.9 nm. Complementarily, Figure 4d illustrates the operating voltage required to achieve on/off ratio over 10. As the area decreased, the operating voltage was reduced, and a low operating voltage of 1.5 V was obtained in the sub‐1 nm regime. Therefore, these results highlight that extreme scaling provides a viable route to low‐voltage, ultrathin FTJ memory suitable for power‐sensitive applications.

**FIGURE 5:**
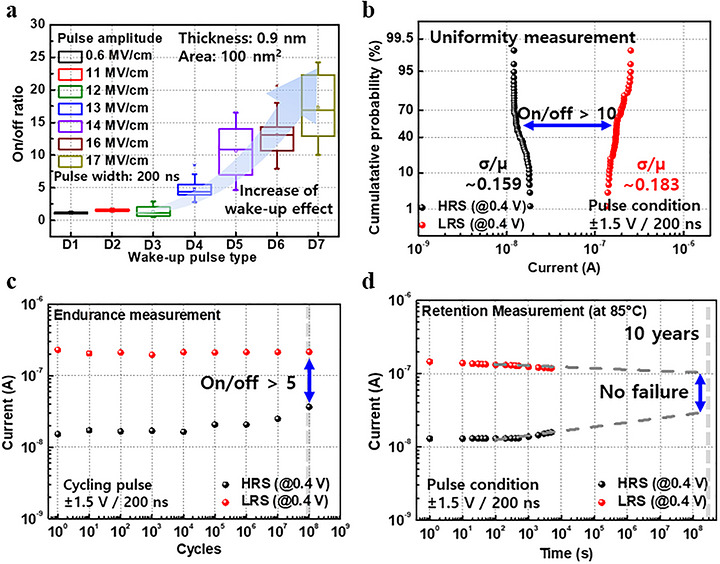
(a) On/off ratios as a function of applied electric field. A clear threshold behavior is seen that the on/off ratio surges above 10 over ∼14 MV cm^−1^, indicating a successful ferroelectric phase activation, whereas below this field the device shows a much smaller TER. (b) Cumulative probability of ON and OFF currents under pulsed operation. The device indicated an average on/off ratio over 10 with pulse amplitude of 1.5 V and pulse width of 200 ns. (c) Endurance characteristics of sub‐1 nm FTJ memory. With electric field cycling utilizing the amplitude of 1.5 V and the width of 200 ns, there is no breakdown after 10^8^ cycles, maintaining on/off ratio over 5. (d) Retention performance at 85°C, showing the stability of the ON and OFF state currents over time after an initial program by +1.5 V or –1.5 V pulse. The device had no overlap of current level by extrapolation.

To elucidate the field‐induced mechanism of the sub‐1 nm FTJ, the device's performance was examined as a function of the applied electric field. Figure 5a plots the on/off ratio after various pulse amplitudes and fixed pulse width (200 ns) of single programming pulse. A clear threshold behavior was observed that a robust on/off ratio exceeding 10 was consistently achieved in the condition of electric field surpassing 14 MV cm^−1^. Fields below this threshold yielded either a very small TER or no discernible memory window, indicating that the ferroelectric phase was not fully activated. This finding reinforces our earlier hypothesis that a sufficiently high electric field is required to overcome the energy barrier between t‐phase and o‐phase in the HZO layer. In essence, low fields produce an incomplete or negligible phase transition, whereas fields above the threshold drive a more comprehensive transformation to the o‐phase, unlocking the full polarization modulation of the tunnel barrier. To confirm the effect of high electric field in detail, current was measured during cycles with pulse amplitude of 1 and 1.5 V (Figure S9). By the wake‐up effect with amplitude of 1 V, pristine device exhibited small on/off ratio. However, sufficient on/ff ratio which is similar to the device with single programming pulse of 1.5 V was obtained after 10^3^ cycles. Based on this result, large electric field can induce large on/off ratio without repetitive pulses. Field‐induced ferroelectricity is further corroborated by polarization‐voltage measurements on a non‐annealed sample as shown in Figure S10, indicating that the electric field itself can nucleate and stabilize the o‐phase without a thermal effect. An unannealed (amorphous) HZO capacitor subjected to a high‐field pulse showed emergent polarization hysteresis, implying that the electric field alone can nucleate and stabilize the o‐phase even without thermal crystallization. Guided by these results, a pulse amplitude of 17 MV cm^−1^ was utilized for further device characterization, as it ensures a fully developed ferroelectric state and a wide memory margin without inducing dielectric breakdown. Under these conditions, the device exhibited high J_on_ with a large on/off ratio, highlighting its suitability for high‐speed read operations.

In addition to electrical characteristics, achieving uniform switching is critical for practical memory array applications. As shown in the current distribution plot in Figure 5b, the device exhibited remarkably narrow distributions for both low resistance state and high resistance state with programming pulse (± 1.5 V / 200 ns) and read pulse (0.4 V / 100 ns). This low variability indicates that field‐induced o‐phase formation is not a rare or filamentary event, but rather a spatially pervasive transformation across the active area once the requisite field is applied. The absence of outlier switching events is important for cross‐point arrays, as it simplifies sensing circuit design and guarantees a clear sensing margin.

Having established these reliable fundamental switching characteristics, the device's reliability was evaluated through rigorous endurance and data retention tests. Read current was measured at 0.4 V in both tests. Endurance characteristics were measured by applying repetitive ±1.5 V / 200 ns pulses, which is sufficient for a suitable on/off ratio as illustrated in Figure S11. The device demonstrated outstanding properties, enduring up to 10^8^ cycles without dielectric breakdown, as depicted in Figure 5c. Throughout the cycling, the memory window remained consistently above 5. There was a gradual, modest reduction in on/off ratio, which could be due to slight fatigue or minor increases in leakage, but the device did not show the abrupt degradation typical of breakdown or shorting. This endurance is outstanding for an ultrathin FTJ, and this stability is a direct consequence of extreme area scaling, which statistically suppresses defect‐assisted leakage paths that typically emerge under electrical stress. The data suggests that further optimization through scaling could enhance the endurance characteristics without compromising the read window.

Furthermore, non‐volatile data retention, especially at elevated temperatures, was assessed. High temperature accelerates any depolarization or drift processes and is a standard condition for retention testing (85°C for 10 years is a common criterion). Figure 5d exhibits the retention characteristics at 85°C after programming pulse (±1.5 V / 200 ns), where the current difference with polarization direction showed minimal degradation over the measured time (10^4^ s range). The distinct current levels allow confident extrapolation to a 10‐year retention period with no overlap, based on established models for non‐volatile memory degradation [50]. The fundamental reason ties back to our earlier points that the MWA process minimized the IL and hence lowered the internal E_dep_. With a high‐quality of ∼0.4 nm IL, there is very little “built‐in” field opposing the polarization during retention. Therefore, polarization can avoid being destabilized even at nanoscale dimensions. Additionally, extreme scaling likely plays a role in retention as well. Any defect‐related leakage that could discharge the ferroelectric over time is greatly reduced in a small area device [51]. Consequently, the ultrathin FTJ is robustly projected to maintain a clear on/off ratio over 10 for a decade at elevated temperature, satisfying non‐volatile memory requirements. Overall, the endurance and retention results underscore that the device is not only high‐performing but also highly reliable, which is critical for high‐density memory deployment.

To establish a comprehensive design guideline for realizing high‐performance FTJs at the ultimate scaling limit, numerical simulations based on a direct tunneling model were conducted [32, 52]. Before presenting the simulation framework, it is important to describe the underlying model and its parameters. The model computes the tunneling current through a dual‐layer barrier (HZO + IL) with an adjustable fraction of HZO assumed to be ferroelectric o‐phase versus non‐ferroelectric t‐phase. These simulations account for the modulation of the potential barrier height influenced by ferroelectric polarization, which enables distinct on and off states in the o‐phase. In contrast, the t‐phase exhibits a static barrier, indicating no change in resistance. Key physical parameters used in the model include assumed potential barrier heights of 1.8 and 2.5 eV at the top and bottom electrodes. Also, a dielectric constant of 25 for HZO was used, and voltage division across the IL was taken into account. In addition to this tunneling mechanism model, the statistical nature of dielectric breakdown was modeled using a Weibull distribution with a shape parameter (β) of 41, in line with our experimental extraction and literature [49]. In our device of 0.9 nm FTJ memory, the o‐phase fraction was calculated to be ∼55% for the stable on/off ratio over 10, consistent with experimental observations of a mixed phase, as shown in Figure S12.

**FIGURE 6:**
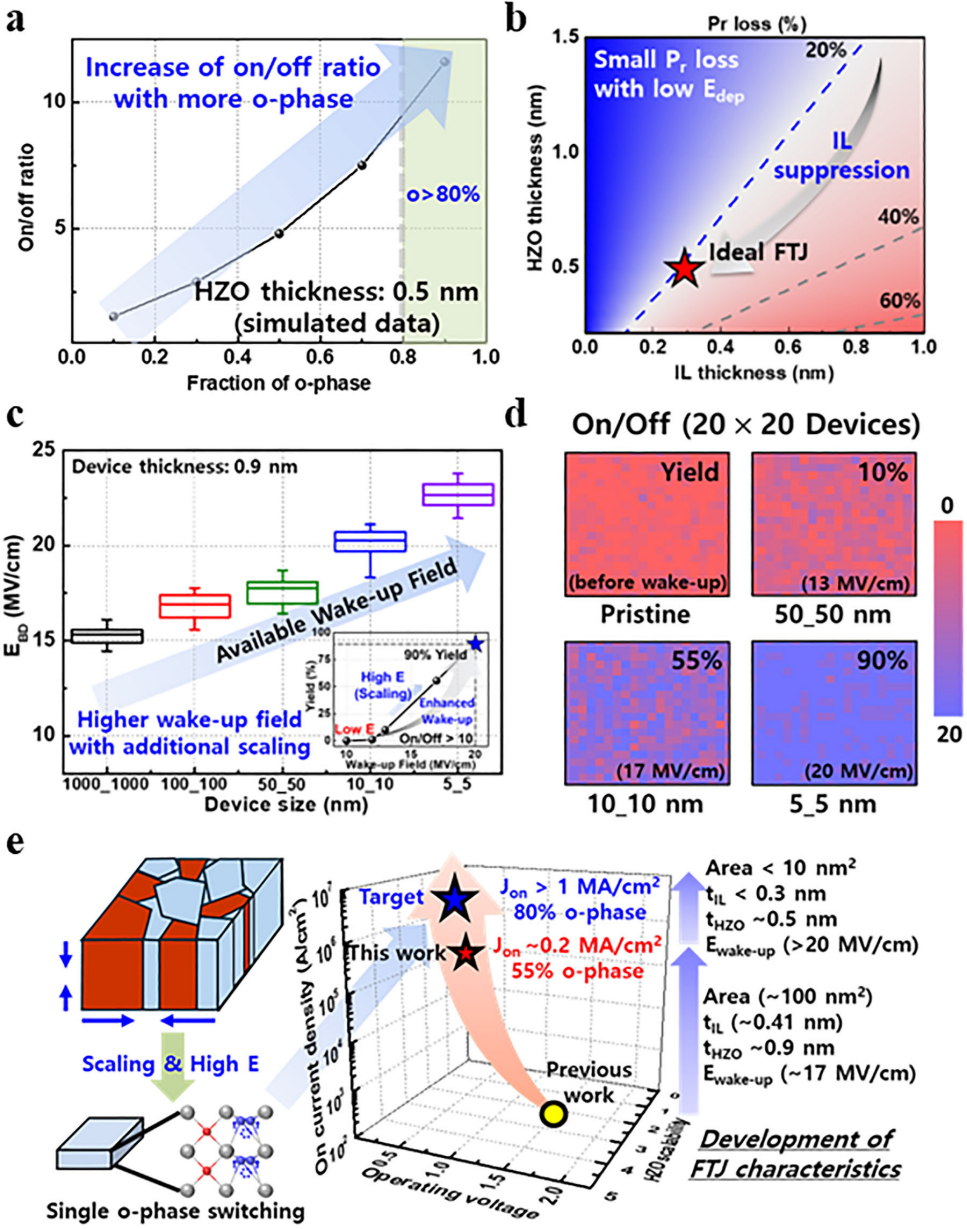
(a) Simulated on/off ratio as a function of the ferroelectric o‐phase fraction in an ideal 0.5 nm HZO film. The simulation indicates that to achieve an on/off >10, the o‐phase fraction must exceed ∼80%. Even a small presence of non‐ferroelectric phase significantly degrades the TER by introducing leakage paths. (b) Calculated polarization loss governed by the E_dep_ as scaling down the thickness of HZO and IL. In ideal FTJ thickness (∼0.5 nm), the IL must be <0.3 nm to keep polarization loss <20% over time. (c) Simulated enhancement of E_BD_ with device area scaling. Aggressive scaling reduces the statistical probability of defects, thereby increasing the tolerable electric field and enabling the use of a higher wake‐up field, which directly translates to a higher yield of functional devices (on/off ratio >10). (d) Yield map of functional cells based on a phase‐distribution model (400 devices). The percentage of achieving on/off >10 is plotted for different wake‐up field amplitudes. At 17 MV cm^−1^, ∼55% of cells become functional, and at 20 MV cm^−1^, ∼90% of cells achieve on/off >10. (e) Roadmap schematic toward the ideal FTJ memory scaling. To approach the theoretical limits (single‐domain switching), the ferroelectric layer needs to be scaled down in thickness (∼0.5 nm) and area (∼1 nm^2^). With ultimate scaling down HZO film and optimized IL condition, single o‐phase‐like switching can be obtained, indicating expected performance of higher J_on_ (>10^6^ A cm^−^^2^) with stable on/off ratio (∼10).

Based on this result, additional simulations were conducted to estimate the FTJ operation at the theoretical thickness limit (∼0.5 nm) of the HZO layer. As shown in Figure 6a, even for an ideal 0.5 nm HZO FTJ, the on/off ratio is critically dependent on the o‐phase fraction. If 50% of the film is o‐phase and the rest remains t‐phase, the simulated on/off ratio is negligible, indicating that the non‐ferroelectric portions act as parallel leakage pathways or a series resistor that shunt the TER effect. The simulation suggests that an o‐phase fraction >80% is required to achieve a respectable on/off >10 in a 0.5 nm device (Figure S13). This highlights that precise phase control, such as high‐field activation, strain, or seed layers, will be crucial to approach single‐unit‐cell ferroelectric devices.

Secondly, interfacial IL control remains a key factor for retention at ultimate scaling. Figure 6b maps the polarization loss as a function of HZO and IL thickness [32]. Accordingly, the effective polarization in the HZO/IL stack was extracted using an electrostatic model that explicitly accounts for the E_dep_ arising from incomplete screening as well as the IL‐induced voltage partitioning, and the polarization loss was defined as the reduction of polarization from the idealized IL‐free, zero‐depolarization limit to the corresponding effective value under these realistic stack conditions. It shows that for an aggressively scaled 0.5 nm HZO film to be viable, the IL thickness must be reduced to below 0.3 nm to maintain polarization loss under 20% while sustaining an o‐phase fraction of ∼80%. Keeping the IL ultrathin can keep the E_dep_ in check to preserve the stored polarization in such a thin ferroelectric. This reinforces our experimental emphasis on IL suppression via MWA.

Thirdly, the role of device area scaling becomes even more indispensable at the extreme limit. Based on the statistical nature of dielectric breakdown and phase distribution model as illustrated in Figures S14 and S15 [53], additional simulations were conducted. Figure 6c shows the simulated enhancement of breakdown field with shrinking area. The tolerable field increases super‐linearly as area decreases, meaning that only by extreme scaling (to a few nm^2^ or a few domains in area) can one reliably apply fields >20 MV cm^−1^ that might be required for single‐domain ferroelectric switching. The profound impact is showcased in the yield maps of 400 devices in Figure 6d, which quantify wake‐up efficiency as a function of the applied field magnitude. To interpret these yield trends in terms of microstructural phase activation, the wake‐up field was translated into a corresponding increase in the effective o‐phase fraction using an empirically fitted, monotonic transformation, where higher fields induce stronger o‐phase activation and the response approaches saturation at sufficiently high fields. In the pristine state and under 15 MV cm^−^^1^, only ∼10% of cells exhibit a clear memory window. However, upon applying a high‐field wake‐up pulse, the yield of functional cells with an on/off ratio over 10 increases sharply to ∼55% at 17 MV cm^−^^1^ and reaches ∼90% at 20 MV cm^−^^1^. These results indicate that aggressive scaling is a prerequisite for enabling high‐field phase activation uniformly across a high‐density memory array.

Synthesizing these numerical simulations and experimental insights suggests a forward‐looking roadmap toward the ideal FTJ device as shown in Figure 6e. This figure charts a clear development trajectory from previous work to our present achievements and sets ambitious targets for future devices capable of single o‐phase switching, which is theoretically ideal for ferroelectric memory. This target can be characterized by a high J_on_ (>1MA cm^−2^) and a stable on/off ratio (>10), with an active area <10 nm^2^, thinner IL thickness (<0.3 nm), theoretically minimized thickness of HZO (∼0.5 nm) [54], and an effective wake‐up field exceeding 20 MV cm^−1^. In addition to area scaling for the high electric field, enhancing overall film uniformity via advanced deposition and annealing techniques can also improve breakdown tolerance [55, 56, 57, 58]. For instance, advanced deposition techniques or interface passivation might further reduce defect densities, raising intrinsic breakdown strength and narrowing the variability in breakdown as shown in Figure S16. Therefore, by advancing extreme device scaling and atomic‐scale materials processing, it is envisioned that the ideal FTJ can be realized, opening a new frontier for non‐volatile memory with unprecedented density, ultra‐low power consumption, and high reliability.

Finally, a performance comparison of HZO‐based FTJ memory devices is summarized in Table 1. The sub‐1 nm FTJ, with extremely scaling and high field‐induced phase transition, exhibits substantial advances, indicating high J_on_ and low operating voltage compared with previous works. With further refinements to IL engineering and device structure as suggested by our simulations, even greater performance can be anticipated in future iterations of this technology.

**TABLE 1:** Performance comparison of HZO based FTJ memory devices.

Structure	Thickness	On/off ratio	On current density	Read voltage	Device area
HZO [32]	4 nm	∼30	∼0.1 A cm^−^^2^	1 V	—
HZO/Al_2_O_3_ [30]	12 nm/1 nm	∼10	5u A cm^−^^2^	2 V	200 µm (diameter)
HZO/TaN [31]	5.5 nm/3 nm	∼100	0.004 A cm^−^^2^	1 V	2.5 × 10^3^ µm^2^
HZO/Al_2_O_3_ [59]	3 nm/1.5 nm	∼5	∼150 A cm^−^^2^	0.5 V	2.5 × 10^5^ µm^2^
AFE‐HZO/Al_2_O_3_ [60]	10 nm/2 nm	∼1500	83 A cm^−^^2^	3.5 V	1.2 × 10^5^ µm^2^
This work: HZO	∼0.9 nm	∼15	∼2 × 10^5^ A cm^−^^2^	0.4 V	10^2^ nm^2^

## 3 Conclusion

In summary, we have demonstrated a synergistic engineering strategy to overcome the inevitable scaling challenges in HZO‐based FTJs, achieving record‐high J_on_ with a sub‐1 nm thickness. The basis of our approach was the strategic decoupling of interfacial and phase stability issues. First, the implementation of low‐temperature MWA proved critical for creating a pristine ferroelectric/electrode interface. This approach effectively suppressed the growth of IL, which minimized interfacial voltage loss and mitigated the detrimental E_dep_. Additionally, the key enabler for unlocking ultimate performance was the extreme scaling of the device area. This strategy statistically enhanced the film's tolerance to dielectric breakdown, permitting the application of a high electric field. This high field was then able to drive a robust and uniform o‐phase formation, even in an atomically thin film. The successful integration combined MWA and field activation by area scaling yielded an unprecedented performance. Sub‐1 nm FTJ devices exhibited a record‐high J_on_ exceeding 10^5^ A cm^−^^2^ with a stable on/off ratio (∼15) at a low read voltage. Furthermore, this robust device demonstrated excellent endurance surpassing 10^8^ cycles and a projected 10‐year data retention. Finally, our modeling results further illuminate a clear pathway for optimizing ferroelectric phase fraction and field effects at near single‐lattice thicknesses. Collectively, this work sets a new benchmark for FTJ performance and offers a comprehensive, scalable manufacturing template for next‐generation high‐density memory. By addressing both materials and device‐level challenges, it opens a viable path for incorporating ferroelectric memory into ultra‐dense, low‐power memory arrays and computing systems, bridging a critical gap in the memory technology landscape.

## 4 Experimental Section

### 4.1 Device Fabrication

A 0.9 nm‐thick HZO layer was deposited by atomic layer deposition (ALD) process using tetrakis (ethylmethylamino) hafnium and tetrakis (ethylmethylaminio) zirconium as precursors. This film was composed of a 1:1 ratio of Hf and Zr and oxidized with ozone for 5 s. The process was conducted on the diced plug‐type wafer to prevent the limitation of uniformity using MWA method. The substrate was maintained at 250°C and the precursor was kept at 90°C for during HZO deposition. A 30 nm‐thick W layer was deposited using radiofrequency sputtering to induce the ferroelectric phase formation via thermal expansion difference. All stacks were deposited on a plug‐type wafer with the bottom electrode of TiN. For aggressive scaling of device area to 100 nm^2^, a 10 nm‐thick TiO_2_ sidewall spacer was deposited by ALD using tetrakis (dimethylamido) titanium precursor at 200°C. Subsequently, the TiO_2_ was then anisotropically etched using a reactive ion etching system with a power of 100 W, utilizing a gas chemistry of 50 sccm CF_4_ and 17 sccm O_2_. This step defines the junction area prior to the final top electrode deposition. Crystallization of the ferroelectric layer was achieved utilizing either rapid thermal annealing (RTA) at 500°C for 1 min in a nitrogen atmosphere or microwave annealing (MWA) at 2 kW for 20 s.

### 4.2 Electrical Measurements

The current–voltage (*I–V*) characteristics of the ultrathin FTJ devices were measured using a semiconductor device parameter analyzer (Keysight B1500A), and pulsed measurements were conducted using a waveform generator/fast measurement unit module. Furthermore, the endurance characteristics with repetitive programming pulse were observed using a Keysight DSOS104A digital storage oscilloscope and Keysight 81160A pulse function arbitrary generator.

### 4.3 Material Characterization

High‐resolution transmission electron microscopy (HR‐TEM) was used to analyze the cross‐sectional structure of the devices. TEM samples were prepared by focused ion beam (FIB) lift‐out and thinning. Imaging was performed on a JEOL JEM‐2200FS.

## Conflicts of Interest

The authors declare no conflicts of interest.

## Supporting information




**Supporting File**: smll73730‐sup‐0001‐SuppMat.docx.

## Data Availability

Research data are not shared.
