# Supplementary material for: Sub‐Nanometer Ferroelectric Tunnel Junctions With Record‐High on‐Current Density Through Synergistic Microwave Annealing and High‐Field Activation
Source: Small. 2026 May 10;22(36):e73730. doi: 10.1002/smll.73730 (PMC13306945; doi:10.1002/smll.73730)
Supplement: Supplementary file 1 — Supporting File: smll73730‐sup‐0001‐SuppMat.docx. [file SMLL-22-e73730-s001.docx]

Supporting Information

Sub-Nanometer Ferroelectric Tunnel Junctions with Record-High On-Current Density through Synergistic Microwave Annealing and High-Field Activation

Layong Jung†, Hojung Jang†, Jongseon Seo, and Hyunsang Hwang^,^^*^

Center for Single Atom-based Semiconductor Device and the Department of Materials Science and Engineering, Pohang University of Science and Technology, Pohang 37673, Republic of Korea.

E-mail: [hwanghs@postech.ac.kr](mailto:hwanghs@postech.ac.kr)


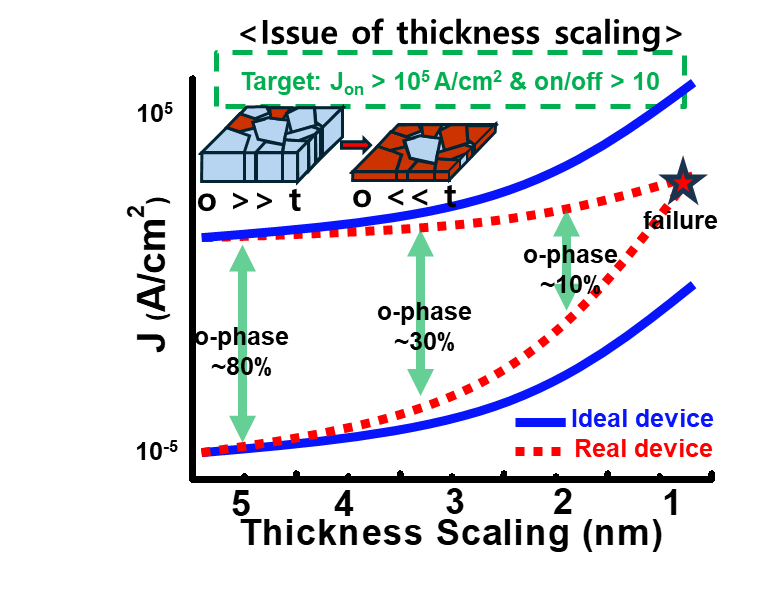


**Figure S1.** Conceptual illustration of how aggressive thickness scaling in Hf_x_Zr_1-x_O_2_ (HZO)-based ferroelectric tunnel junctions (FTJs) leads to a phase imbalance and degrades tunneling electroresistance (TER). As the ferroelectric layer approaches the sub-5 nm regime, surface/size effects favor the non-switchable tetragonal (t) phase at the expense of the orthorhombic (o) phase, thereby weakening polarization and reducing polarization-dependent barrier modulation.^[1-3]^ The resulting decrease in on current density (J_on_) together with an increase in OFF current, arising from both diminished barrier asymmetry and defect-assisted leakage, resulting in small on/off ratio. This schematic explains why prior sub-5-nm devices reported poor windows and highlights the need for strategies that simultaneously stabilize the o-phase and suppress interfacial leakage.


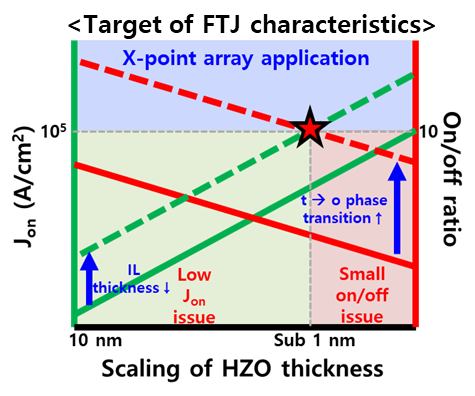


**Figure S2.** Schematic summarizing the two key levers for ideal FTJ operation at ultrathin thickness, which are interfacial layer (IL) control and phase control. The IL, acting as a low-k series element, steals a fraction of the applied voltage, thereby limiting J_on_​.^[4-5]^ In parallel, an insufficient o-phase fraction suppresses polarization-driven barrier modulation, compressing the read memory window (TER). The target device maintains a minimal IL to maximize the internal field in HZO and a high o-phase fraction to maximize TER, enabling low-voltage and high-J_on_​ operation compatible with dense cross-point arrays.


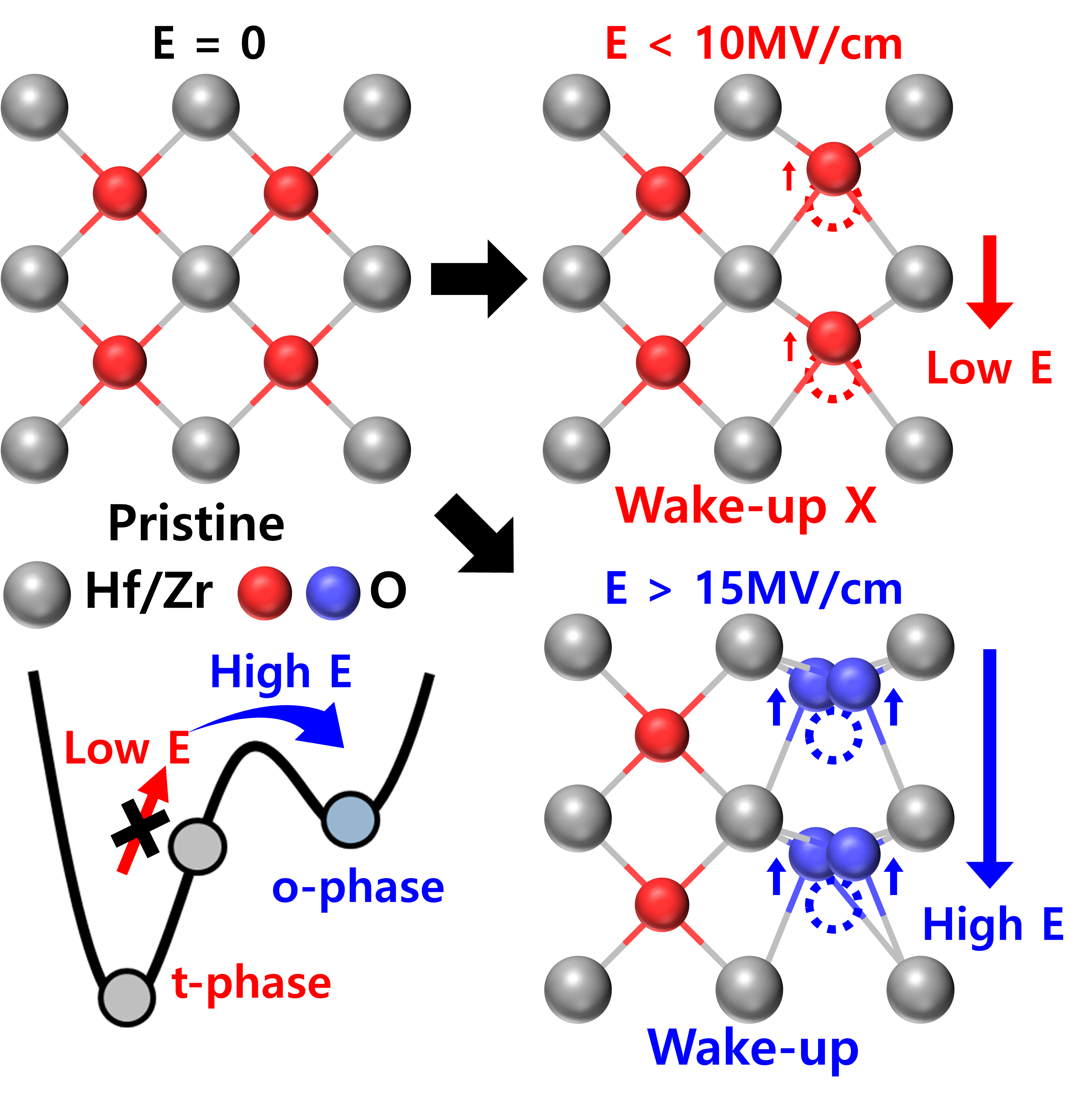


**Figure S3.** Schematic of structural change in ferroelectric layer with increasing the electric field.^[6]^ At low field, the energy supplied is insufficient to overcome the transformation barrier, so the film remains predominantly t-phase with negligible TER. When the effective field exceeds ~15 MV cm⁻¹, field-induced reconfiguration, which is facilitated by oxygen-vacancy bond rearrangements, stabilizes the polar o-phase, increasing the o-phase fraction and strengthening polarization-dependent barrier modulation. This mechanism motivates the combined use of high-field wake-up and area scaling to access robust windows at near-atomic thickness.


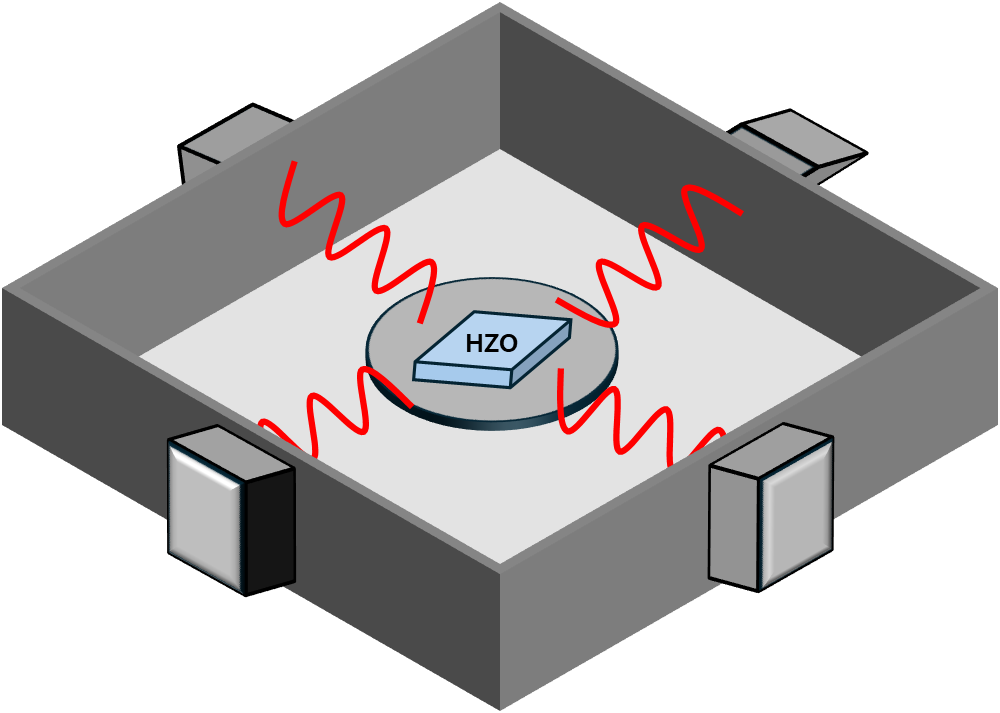


**Figure S4.** Schematic of microwave annealing process illustrating how microwave annealing (MWA) delivers vibrational energy and modest thermal energy directly to the film stack to assist crystallization into the ferroelectric o-phase while reducing global thermal budget compared to rapid thermal annealing (RTA).^[7]^ The localized, rapid energy coupling reduces long-range diffusion and interfacial reactions like electrode oxidation, thereby suppressing IL growth and promoting o-phase formation.^[8]^ This process-only approach preserves stack simplicity and BEOL compatibility while directly addressing voltage-partition losses.


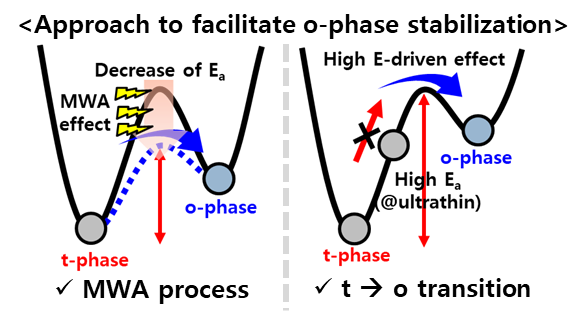


**Figure S5.** Schematic illustration of the method for improving ultrathin FTJ devices. By combining microwave annealing (MWA) with the application of a high electric field, the activation barrier for ferroelectric phase formation can be effectively reduced and overcome, leading to enhanced device performance. The MWA process induces crystallization at lower temperatures, while the applied high field promotes the formation of the o-phase through the wake-up effect. Through these two strategies, sub-nanometer FTJ devices can be operated with sufficient o-phase fraction, resulting in stable on/off ratio.


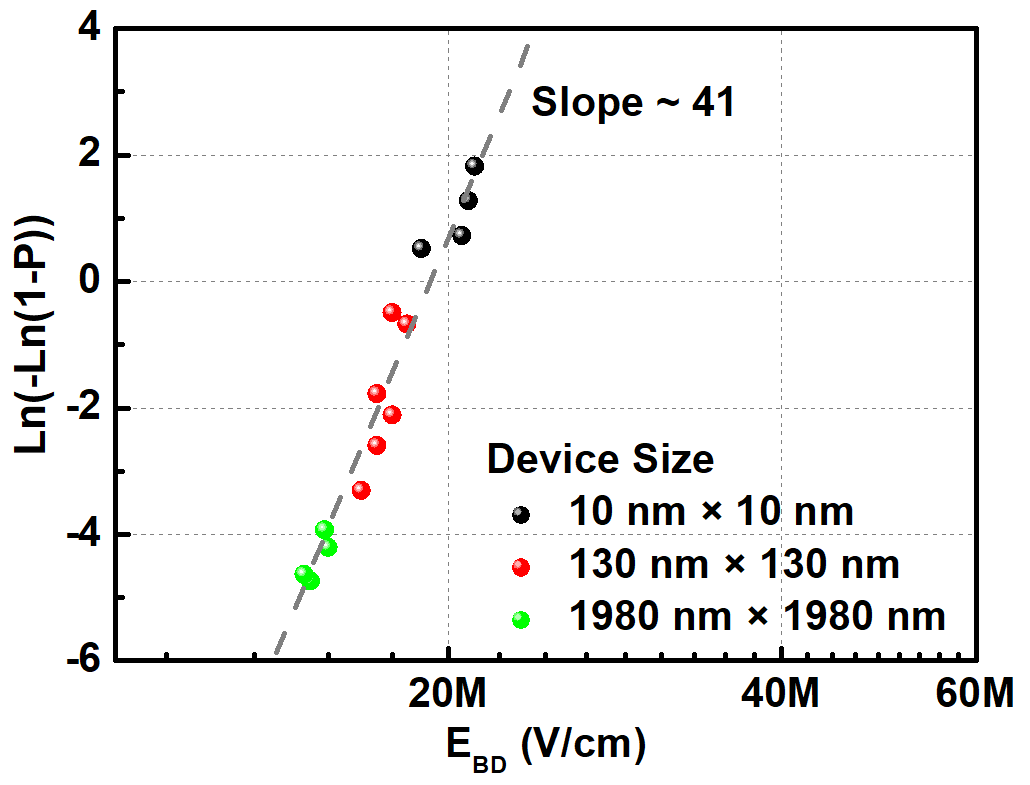


**Figure S6.** Weibull analysis of breakdown field for different device areas to extract β parameter. Weibull plots were generated using the experimentally measured breakdown field distributions for devices with varying active areas. By fitting the data for each device size, a consistent Weibull shape parameter of β ≈ 41 was obtained, indicating that the statistical dispersion of breakdown fields is largely independent of device area within the experimentally accessible range. In contrast, the characteristic breakdown field systematically shifts to higher values as the device area decreases. This analysis confirms that area scaling primarily affects the characteristic breakdown strength rather than the dispersion, thereby providing a statistically basis for extrapolating breakdown behavior to ultrascaled device dimensions.


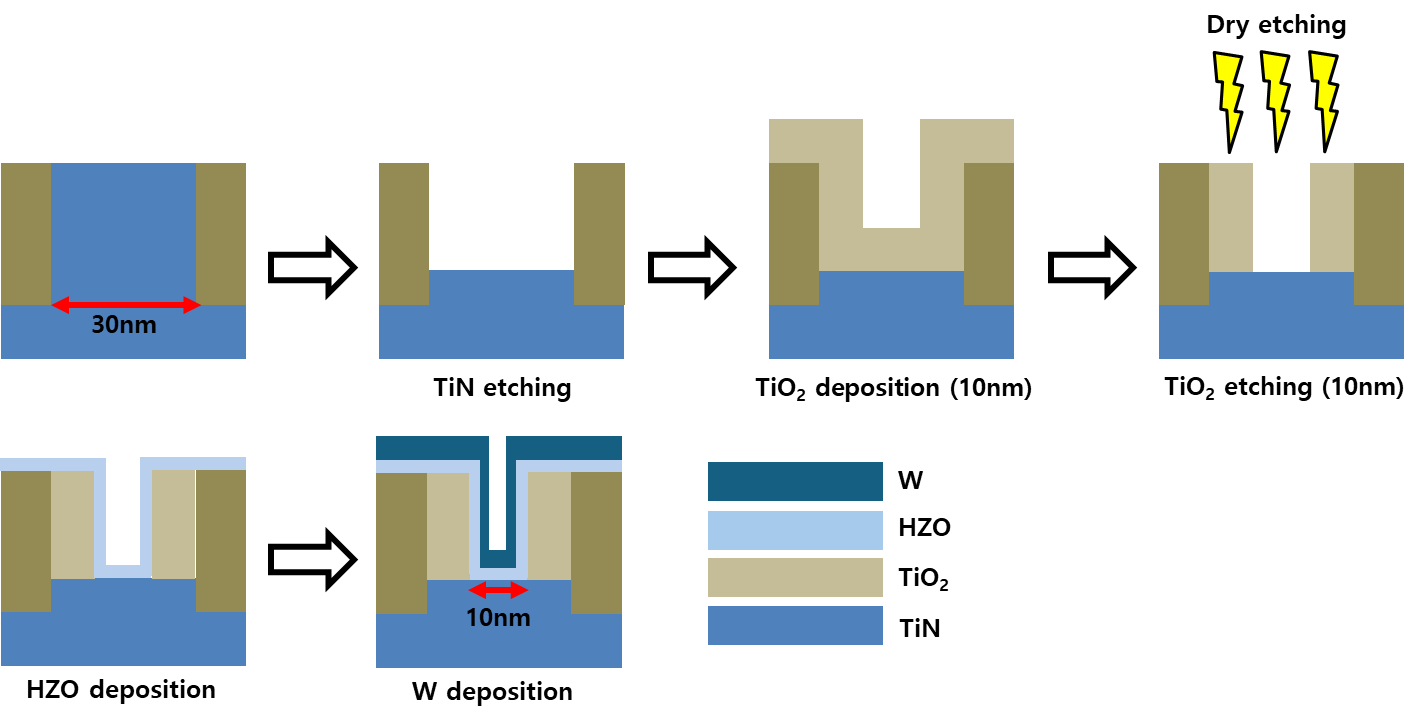


**Figure S7.** Process flow for the fabrication of sub-1 nm FTJs with sidewall structure. To achieve extremely scaled device areas, a 10 nm layer of TiO_2_ was deposited using atomic layer deposition equipment and eliminated using reactive ion etching system. After etching, the residual TiO_2_ sidewall served as a barrier, reducing the effective device area to ~100 nm^2^. This sidewall-constrained scaling enhanced breakdown tolerance by statistically suppressing defect-driven failure, thereby allowing the application of higher electric fields to further increase the o-phase fraction and stabilize device switching characteristics.


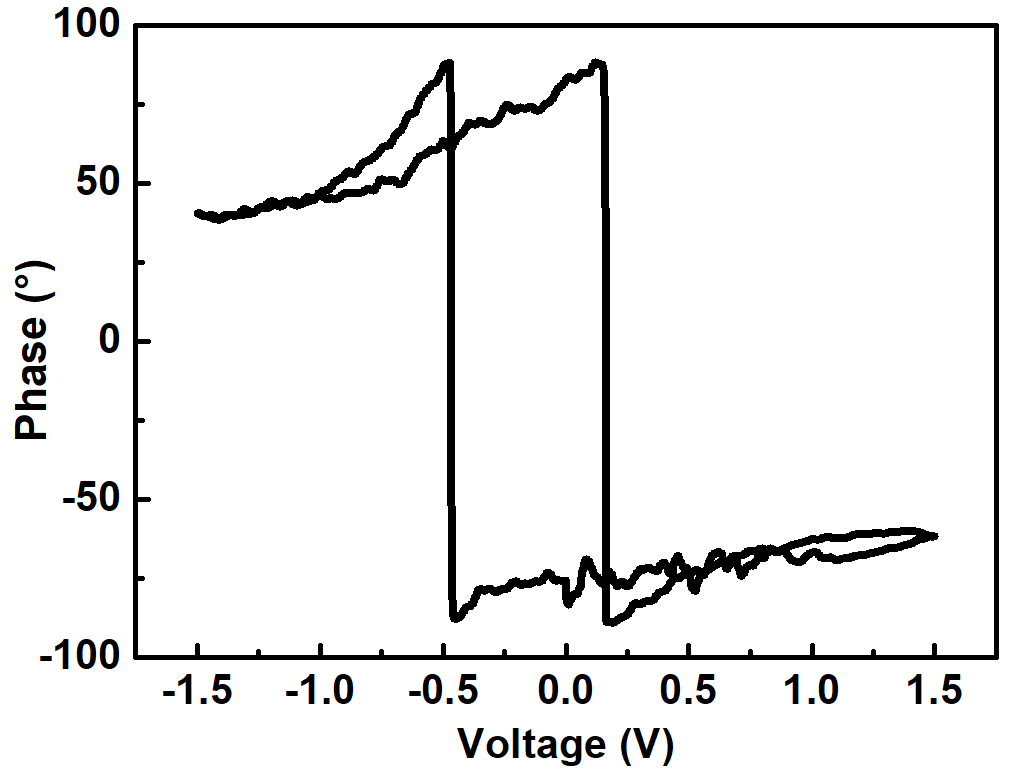


**Figure S8.** Piezoresponse Force Microscopy (PFM) characterization of the HZO ferroelectric layer. PFM measurements were performed to directly verify the ferroelectric nature of the crystallized HZO layer used in this work. The PFM phase and amplitude responses exhibit clear contrast reversal under opposite bias conditions, confirming the presence of switchable polarization in the HZO film. This behavior is absent in the amorphous HZO samples, further supporting that ferroelectric switching emerges only after crystallization. These results provide independent experimental evidence that the observed on/off characteristics in the FTJ devices originate from ferroelectric polarization switching rather than from filamentary or resistive conduction mechanisms.

*
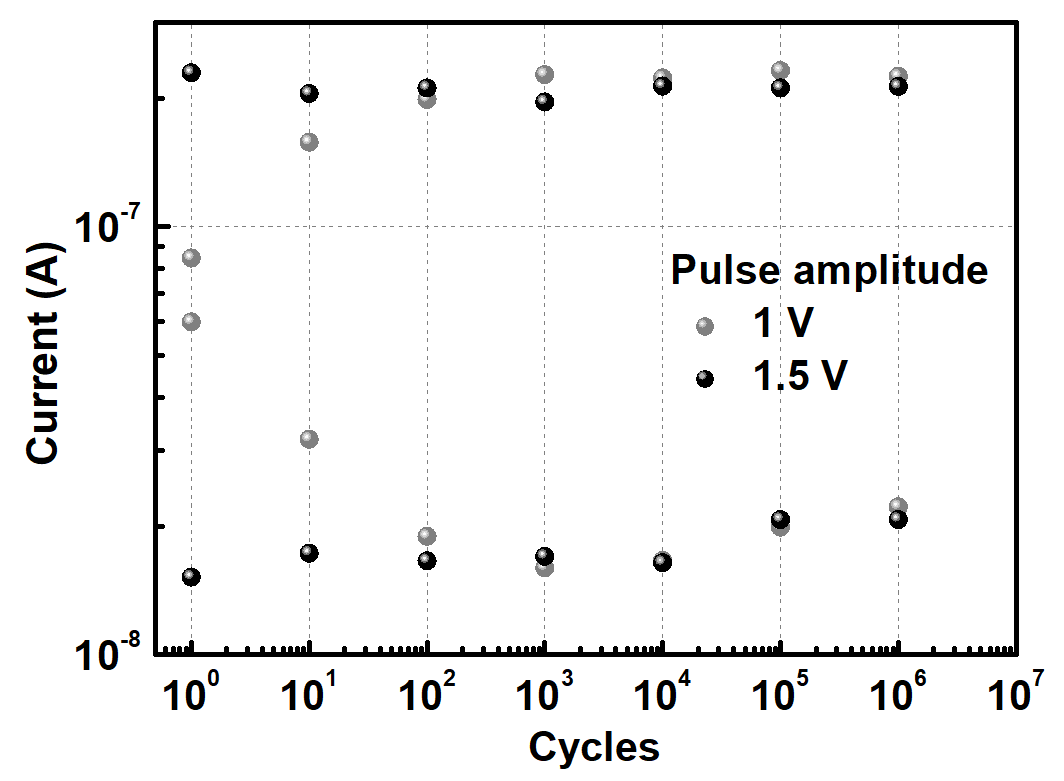
*

**Figure S9.** Wake-up behavior of the FTJ device under low electric field. The on and of current as a function of cycling under different pulse amplitude. When a low pulse amplitude of 1 V was applied, the device initially exhibited a limited on/off ratio. As the number of cycles increased, a gradual enhancement of the on/off ratio was observed, which is attributed to the wake-up effect in the ferroelectric HZO layer. After approximately 10³ cycles, an on/off ratio exceeding 10 was achieved. In contrast, when a higher pulse amplitude of 1.5 V was applied, a sufficiently large electric field was immediately imposed across the ferroelectric layer, resulting in a high on/off ratio from the first cycle without the need for repeated wake-up cycling. These results clearly indicate that the wake-up process strongly depends on the applied electric field, and that higher programming fields can effectively activate ferroelectric polarization switching in a single cycle.

**Figure S10.** Polarization-voltage (P-V) characteristics of a 10 nm HZO capacitor measured without thermal annealing. A bipolar pulse of ±6 V was applied for 30 s, after which the device exhibited ferroelectric hysteresis, confirming that electric field alone can nucleate and stabilize the ferroelectric o-phase in HZO in the absence of a thermal budget. The extracted 2P_r_ ~10 μC cm^-2^ indicates a substantial switchable polarization consistent with field-assisted t- to o- transformation. Notably, this measurement was performed on a large-area of 10^4^ μm^2^ and applying higher fields to smaller junctions is expected to further increase the o-phase fraction, thereby amplifying the polarization signature. These observations support the central mechanism of field-induced ferroelectric activation and motivate area scaling as a practical route to stronger wake-up and larger memory windows.


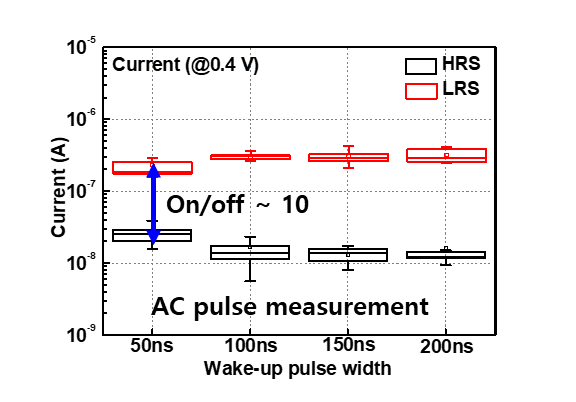


**Figure S11.** On and off currents at the read voltage for various pulse widths. With pulse amplitude of 1.5 V and widths ranging from 50 ns to 200 ns, the device exhibited a stable on/off ratio over 10. As increasing pulse width, larger on/off ratio was obtained. Notably, even at the pulse width of 50 ns, FTJ memory exhibited sufficient on/off ratio, indicating fast switching speed for stable operation in ultrathin thickness.


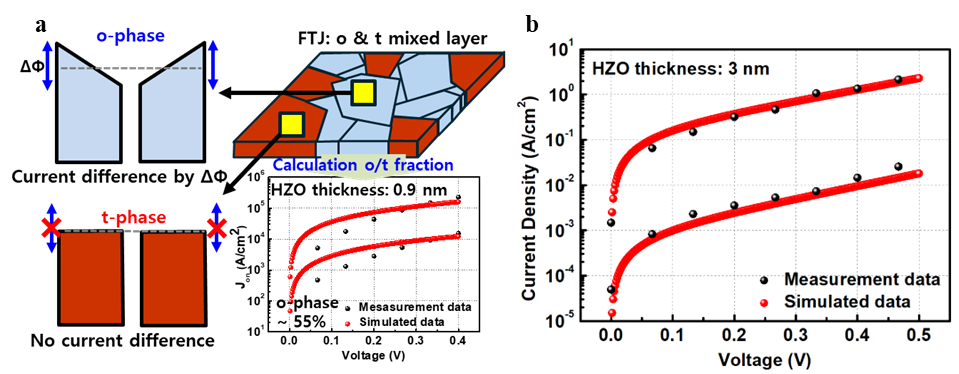


**Figure S12.** a) Schematic of tunnelling mechanism in ultrathin FTJ memory (0.9 nm) and simulated data using a direct tunnelling model.^[9-10]^ b) Fitted data with thicker film thickness (3 nm) to improve the reliability of simulation. Ferroelectric HZO in the FTJ is expected to contain mixed t-phase and o-phase regions, where only the o-phase contributes switchable polarization that modulates the tunneling barrier. To estimate the o-phase fraction in the fabricated FTJ, we used a phase-mixture model anchored to literature values by assigning 2P_r_=70μC/cm² for 100% o-phase and 2P_r_=1μC/cm² for t-phase.^[11]^ Subsequently, an effective P_r_ was extracted from the measured on/off ratio to calculate the corresponding o-phase fraction. Based on this framework, numerical simulations using a direct tunneling model were conducted to evaluate the behavior of ideally scaled devices. In thinner thickness, alternative mechanisms such as Fowler Nordheim tunnelling, trap assisted tunneling and thermionic emission do not match the experimental data.^[12-13]^ These simulations accounted for the modulation of the potential barrier height influenced by ferroelectric polarization, which enables distinct on and off states in the o-phase induced by ferroelectric polarization. In contrast, the t-phase exhibits a static barrier, preventing sufficient memory window. Moreover, based on the thickness and dielectric constant of HZO and IL by TEM analysis, voltage division was considered in the simulation. For the simulation with direct tunnelling equation as followed,

$$J_{DT}=C\frac{\exp\left[ \alpha\left\{ \left( \Phi_{2}-\frac{qV}{2} \right)^{3/2}-\left( \Phi_{1}+\frac{qV}{2} \right)^{3/2} \right\} \right]}{\alpha^{2}\left[ \sqrt{\Phi_{2}-\frac{qV}{2}}-\sqrt{\Phi_{1}+\frac{qV}{2}} \right]^{2}}$$

$$\times\sinh\left[ \frac{3qV}{2}\alpha\left\{ \sqrt{\Phi_{2}-\frac{qV}{2}}-\sqrt{\Phi_{1}+\frac{qV}{2}} \right\} \right]$$

the parameter was estimated. The potential barrier height was 1.8 eV and 2.7 eV, dielectric constants of 25 for HZO and 20 for IL. Furthermore, an effective mass was 0.3 m_0_ in HZO. By applying this model to the experimentally measured DC current-voltage characteristics, the o-phase fraction was estimated to be approximately 55% assuming a maximum polarization value of 70 μC cm^-2^.


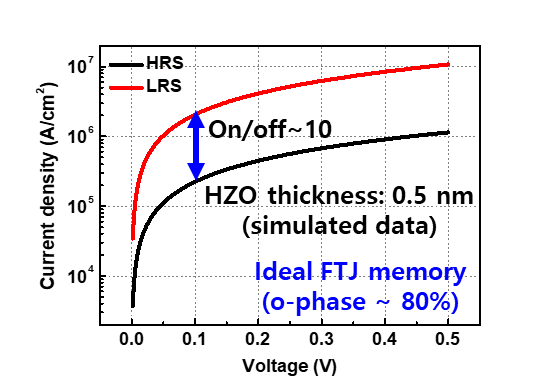


**Figure S13.** Simulated FTJ current at an HZO thickness of 0.5 nm using parameters calibrated to the 0.9 nm device. The analysis shows that an o-phase fraction ≥~80% is required to sustain on/off ratio >10 at the theoretical thickness limit, highlighting phase purity as the foremost requirement for functional FTJs at atomic thickness and motivating high-field activation to maximize the ferroelectric fraction.


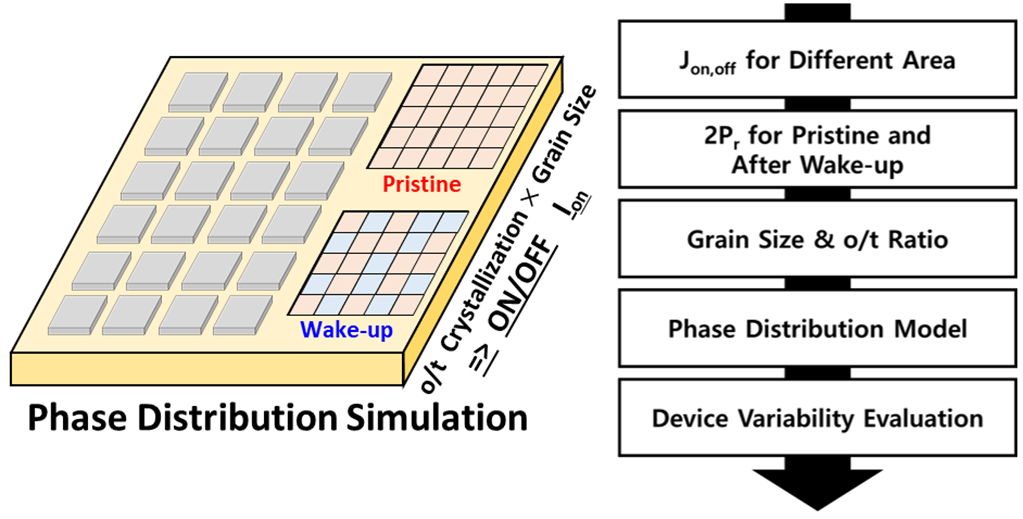


**Figure S14.** Simulation flow of the phase distribution model based on the direct tunneling mechanism.^[14]^ This figure summarizes the simulation workflow used to interpret the area-dependent device behavior and its evolution after wake-up. The model is based on a direct-tunneling mechanism, where polarization in HZO modulates the effective tunneling barrier profile between the top and bottom electrodes, producing a current contrast between the ON and OFF states. From the experimental FTJ data (0.9 nm HZO), an effective polarization-related parameter is inferred from the measured on/off ratio using the direct-tunneling model and converted into an estimated o-phase areal fraction using a phase-mixture assumption between the o-phase and t-phase limits. This conversion is applied on a device-by-device basis to translate the electrical dispersion into a statistical distribution of o-phase fraction for each device area, thereby capturing device-to-device variability. Wake-up-induced phase activation is incorporated by mapping the wake-up field to an increase in o-phase fraction through an empirically fitted monotonic transformation, where higher fields lead to stronger o-phase activation with threshold-like increase and eventual saturation. The fitted mapping is calibrated to reproduce the experimentally observed yield trend in Fig. 6d, and the resulting o-phase distributions are used to simulate area-dependent yield statistics under different wake-up fields.


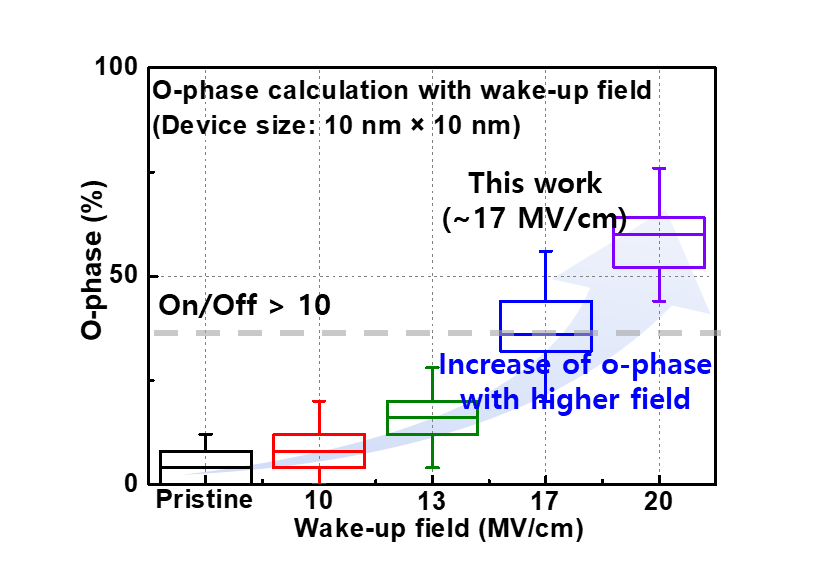


**Figure S15.** Simulated o-phase distribution with increasing wake-up field. The graph presents the o-phase ratio of the devices as a function of the wake-up field, indicating that higher wake-up fields are required to achieve stable performance. The o-phase fraction increased with higher wake-up field, leading to higher on/off ratio. However, the device in this work can be reliably operated up to 17 MV cm⁻¹ due to the dielectric breakdown issue. Therefore, achieving wake-up fields beyond 17 MV cm⁻¹ requires further area scaling or IL suppression to shift the breakdown distribution and widen the stable high-field window. These results quantitatively link wake-up field to phase composition and provide a useful design lever for inducing ultrathin FTJs toward the pure o-phase ideal.


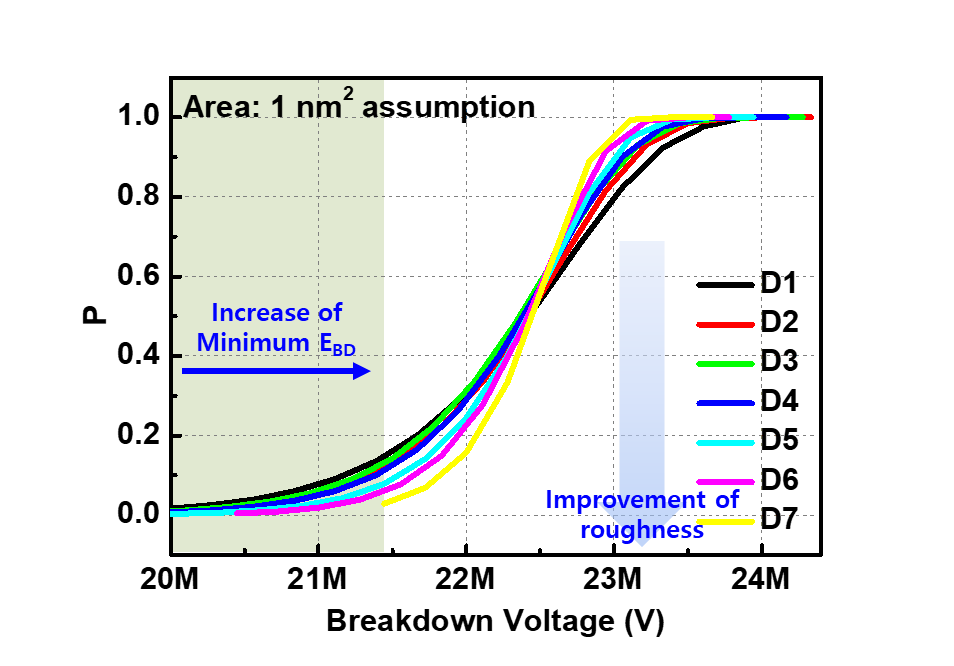


**Figure S16.** Weibull distribution of the breakdown voltage for an active area of 1 nm^2^ with simulated data. The parameter of roughness was controlled from D1 to D7. D1 has the lowest β and D7 has the highest β. The beta values were plotted by increasing from 26 to 56 in steps of 5. A higher β indicates improved roughness, resulting in the narrow distribution of breakdown voltage. Therefore, with improved roughness, ultrathin HZO exhibited enhanced breakdown tolerance, enabling higher electric field for the o-phase formation. Simulated breakdown-voltage distributions for an active area of 1 nm², analyzed using a Weibull model to quantify the impact of interface/film roughness. The roughness parameter is swept from D1 → D7, where D1 corresponds to the poorest morphology and yields the lowest shape factor β, while D7 represents the smoothest case with the highest β. In the Weibull formalism, a larger β indicates a tighter statistical spread (improved reliability), and the distribution follows,^[15]^

$$P_{BD}\left( E \right)=1-exp\left[ -\left( \frac{E}{E_{0}} \right)^{\beta} \right],$$

with E_0_​ which is the characteristic of scale field. As roughness improves (D1→D7), the slope of the Weibull plot increases and percentile breakdown fields shift to higher values, reflecting suppression of defect clusters that nucleate premature failure. Consequently, ultrathin HZO exhibits enhanced breakdown tolerance, expanding the safe high-field window needed to drive o-phase formation during wake-up. This analysis provides a quantitative reliability target, morphology/roughness control, to complement area scaling in accessing >17~20 MV cm⁻¹ programming fields at the near-atomic thickness limit.

**Reference**

[1] M. H. Park, H. J. Kim, Y. J. Kim, Y. H. Lee, T. Moon, K. D. Kim, S. D. Hyun, C. S. Hwang, *Applied Physics Letters* **2015**, 107.

[2] M. Lederer, S. Abdulazhanov, R. Olivo, D. Lehninger, T. Kämpfe, K. Seidel, L. M. Eng, Scientific reports **2021**, 11, 22266.

[3] Z. Liu, X. Shi, J. Wang, H. Huang, npj Quantum Materials **2024**, 9, 44.

[4] Y. Park, J. Kim, S. Kim, D. Kim, W. Shim, S. Kim, Journal of Materials Chemistry C **2023**, 11, 13886.

[5] H. Ryu, H. Wu, F. Rao, W. Zhu, Scientific reports **2019**, 9, 20383.

[6] D. Zhou, J. Xu, Q. Li, Y. Guan, F. Cao, X. Dong, J. Müller, T. Schenk, U. Schröder, Applied Physics Letters **2013**, 103.

[7] B. Zhao, Y. Yan, J. Bi, G. Xu, Y. Xu, X. Yang, L. Fan, M. Liu, Nanomaterials **2022**, 12, 3001.

[8] T. Jung, H. Shin, J. Ahn, S. Jeon, IEEE Transactions on Electron Devices **2025**, 72, 3076.

[9] J. Hwang, Y. Goh, S. Jeon, Small **2024**, 20, 2305271.

[10] J. Yoon, S. Hong, Y. W. Song, J.-H. Ahn, S.-E. Ahn, Applied Physics Letters **2019**, 115.

[11] W. Wei, X. Ma, J. Wu, F. Wang, X. Zhan, Y. Li, J. Chen, Applied Physics Letters **2019**, 115.

[12] M. Kobayashi, Y. Tagawa, F. Mo, T. Saraya, T. Hiramoto, IEEE Journal of the Electron Devices Society **2018**, 7, 134.

[13] Q. Luo, Y. Cheng, J. Yang, R. Cao, H. Ma, Y. Yang, R. Huang, W. Wei, Y. Zheng, T. Gong, Nature communications **2020**, 11, 1391.

[14] H. Jang, A. Kashir, S. Oh, K. Lee, L. Jung, M. Habibi, T. Schenk, H. Hwang, IEEE Electron Device Letters **2024**, 45, 344.

[15] C. Sire, S. Blonkowski, M. J. Gordon, T. Baron, Applied Physics Letters **2007**, 91.
